# Disease knowledge and quality of life among rheumatoid arthritis patients: a cross-sectional study

**DOI:** 10.1186/s41927-025-00523-w

**Published:** 2025-07-01

**Authors:** Lara Albiss, Suhaib Muflih, Bushra Hijazi, Osama Y. Alshogran, Walid Al-Qerem, Manar Abu Khurmah, Ahmad Al-Azayzih, Hani Shatnawi, Yazan Shakatira

**Affiliations:** 1 Department of Clinical Pharmacy, Faculty of Pharmacy, Jordan University of Science and Technology, Irbid, 22110 Jordan; 2 Department of Clinical Pharmacy, Faculty of Pharmacy, AlZaytoonah University of Jordan, Amman, 11733 Jordan; 3 Princess Basma Hospital, Outpatient Clinics, Irbid, 21110 Jordan

**Keywords:** Rheumatoid arthritis, Disease knowledge, Quality of life, Severity of disease

## Abstract

**Objectives:**

This study aimed to predict the knowledge of disease, quality of life, and related factors among rheumatoid arthritis patients.

**Methodology:**

In this cross-sectional study, a total of 225 participants were recruited by convenience sampling from the RA outpatient clinics at Princess Basma Hospital and King Abdullah University Hospital in the north of Jordan between October 2023 and January 2024. The knowledge of RA was assessed using the adapted Rheumatoid Arthritis Knowledge Assessment Scale (RAKAS). Health-related quality of life (HRQoL) was evaluated using the generic EQ-5D-3 L instrument. Disease activity and remission were measured by DAS-28 ESR, which involved patient global assessment, ESR, and the number of swollen and tender joints. Data collection was achieved by face-to-face interviews and reviewing medical records. Predictors of disease knowledge and QoL were identified using quantile regression, One-way ANOVA, and multiple linear regression.

**Results:**

The mean age of participants was 51.9 years, with 86.2% being female. Only 9.3% and 20.9% of patients, respectively, had “poor” or “low” knowledge, while 42.7% and 27.1% of patients, respectively, had " adequate " or “excellent” knowledge. Significant correlations of RA knowledge were observed with age, education level, duration of RA, and income. Specifically, younger patients, those with longer disease duration, higher education levels, and higher income demonstrated better knowledge of RA. Income and DAS score were significantly associated with the utility. Higher income levels were associated with an increase in utility. There was no association between disease knowledge and QoL in RA patients.

**Conclusion:**

Adequate knowledge of the disease is prevalent among RA patients. Education level significantly affected both disease knowledge and quality of life. Interventions to enhance patient education and proper medication management are essential to improve health outcomes.

**Clinical trial number:**

Not applicable.

## Introduction

Rheumatoid arthritis is a common chronic autoimmune disease affecting healthy tissues in the body [1], especially the synovial membrane lining, leading to inflammation, swelling, stiffness, and pain within the joints [2, 3]. RA can lead to multiple complications due to chronic inflammation and multi-organ involvement. Firstly, the clinical presentation primarily includes joint damage as a prominent outcome, often resulting in deformities, loss of function, and disability [4]. Secondly, patients with RA are associated with an elevated risk of cardiovascular diseases, including heart attacks and strokes, compared to patients without RA [5–7]. Furthermore, RA patients using corticosteroids may be more susceptible to osteoporosis-related fractures [8]. Rheumatoid nodules, which are solid lumps that develop beneath the skin around the affected joints, are another possible complication [4]. However, RA can cause interstitial lung disease, which affects the lungs [9]. RA can also result in symptoms of systemic inflammation, such as weight loss, fatigue, and fever [4]. Inadequately managed RA symptoms can have a major impact on their functional mobility, disability, and ultimately, quality of life [2, 10]. Therefore, patients’ knowledge about the R disease can play a key role in minimizing symptoms, preventing complications, and enhancing the overall health-related quality of life (HR-QoL) [11]. Patient education has been defined as “a planned combination of learning activities designed to help people with disease or illness make changes conducive to health [12]. Clearly understanding health conditions can help patients adhere to prescribed medications, recognize potential adverse reactions, and seek appropriate medical assistance when necessary [13]. Previous studies demonstrated the association between disease knowledge among RA patients and medication adherence, patient outcomes, and self-management [14, 15], underlining the urgent need for personalized educational activities to successfully address knowledge gaps and enhance HR-QoL [14, 16]. Moreover, a multifaceted approach study, utilizing informative pamphlets, interactive workshops, and digital resources, showed a remarkable improvement in patients’ understanding of RA treatment options and recognizing early disease symptoms [15]. Furthermore, Song et al.‘s (2020) study reported the significance of continued education in improving medication adherence, as well as higher QoL and self-efficacy in managing RA [17]. Digital health interventions such as mobile applications and online resources have a significant impact on patient knowledge of RA and its management [18].

These findings underscore the paramount importance of patient education in enhancing disease management and QoL. Previous studies in Jordan had focused on reporting the prevalence of RA, disease severity, and associated comorbidities [19, 20]. A limited number of studies, however, investigated the knowledge of RA and its associated factors in Jordan. Therefore, the current study was conducted using a validated survey tool to evaluate patient knowledge about RA disease and QoL in Jordan.

## Materials and methods

### Study design and settings

This cross-sectional study was carried out among RA patients. The sample was conveniently collected from the tertiary referral hospitals serving the public in northern Jordan, the RA outpatient clinics at Princess Basma Hospital and King Abdullah University Hospital (KAUH). Subjects were approached through face-to-face interviews and informed about the study objectives. The study was conducted from October 2023 to January 2024. Ethical approval to conduct the study was acquired from the International Review Board (IRB) of KAUH )182/2023), the Jordan University of Science and Technology (JUST), and the Ministry of Health (MOH). Before the participant’s enrolment in this study, written informed consent was taken. All data was kept confidential.

Patients who were 18 years of age or older, visited the rheumatology outpatient clinic, and took at least one RA medication were qualified to participate in this study. Exclusion criteria included people attending the clinic for diagnostic purposes, cancer chemotherapy patients, other types of arthritis, transplanted patients, and pregnant women.

### Data collection and instruments

Face-to-face interviews were conducted by a well-trained pharmacist to fill out a structured questionnaire after receiving the consent form from eligible participants. No incentives or compensation were offered to the participants to avoid response bias. A rheumatologist was also involved in collecting measures about the DAS score using the DAS-28 ESR. The first part of the questionnaire encompasses participants’ socio-demographic characteristics, medical history, and lifestyle. The second part of the questionnaire was adopted from previously published work that assessed knowledge of RA using the adapted Rheumatoid Arthritis Knowledge Assessment Scale (RAKAS), a validated novel instrument developed to document the extent of disease knowledge in patients with RA [21]. The third part included an assessment of HRQoL using the EQ-5D-3 L instrument developed by Euro QOL Group [22].

### Rheumatoid arthritis knowledge assessment scale

A validated novel instrument was previously developed to document the extent of disease knowledge in patients with RA [21]. We translated this tool by the backward approach. This tool had a high response rate, better internal consistency, and established its efficacy by demonstrating high specificity and sensitivity [21]. The calculation of the RAKAS score involved assigning a (+ 1) for each correct response and a (0) for each incorrect response. The cumulative score, obtained by summing the scores for each item of the tool (items 1–13), was regarded as the final score. A patient who correctly answered more than 70% of the questions will be considered to have excellent knowledge. If a patient correctly answered between 50% and 70% answers, it would be assigned to have adequate knowledge. The patient would be considered to have low knowledge if the percentage of correctly answered questions was between 30% and 50%, and poor knowledge if the answers were less than 30%. The tool’s final draft had a maximum of 14 points. According to the previous criteria, a patient was considered to have excellent knowledge if their score was 11 or higher, and adequately knowledgeable if their score was between 8 and 10. A score of 5 to 7 was considered low knowledge, and a score of 4 or less indicated poor knowledge [21]. During pilot testing, the RAKAS scale’s understandability and readability were evaluated, and its internal consistency (Cronbach’s alpha) was found to be greater than 0.7. However, because the Arabic-translated version of the RAKAS tool has been adapted, additional research in the MENA region should be encouraged to support the tool’s cross-cultural validity.

### HRQoL using the generic EQ-5D-3L instrument

The first part of the instrument is a description of the patient’s HRQoL in terms of the following five dimensions: mobility, self-care, usual activities, pain/discomfort, and anxiety/depression. The answers for each dimension were rated on a three-level scale of no problems, some problems, and unable to/extreme problems. The EQ-5D-3 L responses collected were scored to calculate the utility index value using the United Kingdom general population value sets (the crosswalk approach) [22]. While disease-specific measures for RA-QoL are available, this study did not intend to monitor or evaluate the effect of treatment or the progress of RA patients’, thus a generic QoL (i.e., EQ-5D-3 L) was used to measure RA patients’ quality of life, which could allow for comparing the QoL of RA patients with those with other chronic conditions [23].

### DAS-28-ESR score

It is a clinical measure that assesses disease activity and remission in patients with rheumatoid arthritis by using patient global assessment, ESR, and the number of swollen and tender joints [24].

### Statistical analysis and sample size

This study involved a minimum sample of 377 patients obtained using Epi Info 7.0 and Raosoft calculators. The two-sided confidence level of 95%, a desired statistical power (1 – β) of 80%, and a margin of error of 5% were used to estimate the sample size. According to the American College of Rheumatology (ACR) criteria, the expected prevalence of RA patients is reported to be up to 1.25% [25]. However, Jordan reported a lower RA prevalence of 0.36% [26], using a sample size calculator relies on the prevalence of the RA (0.36%) and the precision of 0.01 (or margin of error), and the Zα/2 for alpha 0.05 (= 1.96) a sample of 137 was needed [27]. Throughout the data collection period, 225 voluntarily agreed to participate in this study. The study population consisted of all RA patients who fulfilled the requirements of the American College of Rheumatology (ACR)–European League Against Rheumatism (EULAR) 2010 criteria. The statistical package IBM SPSS version 26.0 was used in data analysis. Normality was tested first by the Shapiro-Wilk test, eye inspection of the Q-Q plot, and a histogram with normal curves; based on these tests data was not normally distributed; therefore, a nonparametric test was conducted. Descriptive statistics were used to describe the research participants’ demographic and clinical data. Continuous variables were represented by means, ranges, and standard deviations, while categorical variables were represented by frequencies and percentages. For RAKAS knowledge score, the normality was tested first by the Shapiro-Wilk test, eye inspection of the Q-Q plot, and a histogram with normal curves, based on these tests data were not normally distributed (*p* < 0.05); therefore, nonparametric test was conducted, and quantile regression was used to analyze the data. In contrast to the One-way ANOVA and the Ordinary least squares (OLS) regression, Quantile regression and the Kruskal-Wallis test do not assume normality. Therefore, the Quantile regression was used for the RAKAS, and the Kruskal-Wallis test was used to assess the relationship between QoL_Utility and other variables due to a lack of normality assumption. However, the significant variables and those approaching significance were introduced into linear modeling after transforming the QoL_Utility, using a mathematical function (i.e., ldl.Normal) to obtain normally distributed data. While using the Kruskal-Wallis, the subcategories “Remission” and “Mild” disease activities of DAS28 were combined into the one subcategory remission/mild due to the small number of observations in each subcategory, as well as to avoid false negatives that could result from many multiple comparisons. Next, a linear regression was performed after testing the assumption of multicollinearity, in which VIF was less than 10, indicating that there is no correlation between the independent variables in a multiple regression model (see Tables 1 and 2).

**Table 1:** The relationship between RA participants’ quality of life (Eq. 5D) and variables of interest. Using the Mann-Whitney U test Anf Kruskal-wallis test

Variables		Mean rank	*P*-value
Gender			
	Male	128.81	0.145
	Female	110.47	
Age			
	< 40	141.2	0.079
	40–50	116.72	
	50–60	101.93	
	> 60	110.48	
Education			
	Primary	90.28	0.007
	Secondary	114.65	
	Diploma	119.04	
	Bachelors or Higher	137.85	
BMI			
	Normal	116.32	0.562
	Overweight	117.86	
	Obese	107.39	
Income			
	< JOD 500	101.03	0.003
	≥JOD 500	126.92	
Number of Comorbidities			
	RA alone	123.52	0.103
	RA + 1	115.8	
	RA + 2	109.28	
	RA + 3	95.34	
RAKAS			
	Poor	88.31	0.336
	Low	116.94	
	Adequate	115.18	
	Excellent	115.03	
Employment			
	Unemployed	109.33	0.057
	Employed	131.64	
DAS28			
	Remission/Mild	128.97	0.001
	Moderate	107.73	
	Severe	69.29	
Duration of RA			
	< 5 years	118.29	0.225
	5–10 years	117.36	
	> 10 years	101.76	

*Significant level < 0.05; BMI: Body Mass Index; RAKAS: Rheumatoid Arthritis Knowledge Assessment Score; DAS28: Disease Activity Score

**Table 2:** Multiple linear regression model of predictive variables associated with participants QoL utility

Model		Unstandardized coefficients		Standardized coefficients	t	Sig.	Collinearity statistics	
		B	Std. Error	Beta			Tolerance	VIF
1	(Constant)	0.878	0.125		7.031	0.000		
	Gender	0.014	0.045	0.022	0.301	0.764	0.835	1.197
	Age	− 0.013	0.019	− 0.053	− 0.659	0.511	0.674	1.484
	Education	0.026	0.017	0.114	1.572	0.118	0.814	1.229
	Income	0.068	0.032	0.153	2.133	0.034	0.833	1.200
	Number of Comorbidities	− 0.013	0.015	− 0.070	− 0.890	0.375	0.698	1.433
	Employment	0.005	0.044	0.008	0.117	0.907	0.834	1.199
	DAS28	− 0.133	0.024	− 0.367	-5.441	0.000	0.948	1.055
	Duration of RA	− 0.038	0.018	− 0.147	-2.182	0.030	0.947	1.056

a. Dependent Variable: Utility_QoL

## Results

### General characteristics of the study participants

This study involved a total of 225 patients (86% females and 14% males), with a mean age of 51.86 (± 11.43). The number of patients who refused to participate in the study was 12, giving a response rate of 94.9%. Reasons for refusal include time constraints, tiredness, or not being interested in participation. The mean BMI was 29.1(± 5.67) Kg/m^2^, and less than half (22.7%) of the study participants had a normal BMI. 78% (*n* = 176) of participants were married, and 21.8% (*n* = 49) were single or divorced. Almost 15% (*n* = 34) of the participants were current smokers. About a third of participants had completed their college and university studies, while 24.4% (*n* = 55) had only completed their primary school, and 42.2% (*n* = 95) had completed secondary school. The majority of participants (83.6%, *n* = 188) were unemployed, and 53.8% (*n* = 121) had a monthly income of less than 500 JD. Most of the participants (*n* = 206) were insured, and 93.3% (*n* = 210) lived with their family. Among the participants, 37.3% (*n* = 84) had RA alone, and 45.8% had RA for less than 5 years.

When participants were asked about comorbidities, hypertension was present in 38.7% (*n* = 87) of them, while 24% (*n* = 54) had diabetes mellitus (DM), and 9.8% (*n* = 22) had other cardiovascular diseases (CVDs). Osteoporosis was found in 24.9% (*n* = 56) of participants, while 12.4% (*n* = 28) had thyroid disease and 11.1% (*n* = 25) had dyslipidemia (see Table 3).

**Table 3:** General characteristics of study participants (*N* = 225)

Variable	Frequency (%)
**N**	225
**Age (mean ± SD**,** Years)** Less than 40 years 40–50 years 50–60 years More than 60 years	51.86 ± 11.43 23(10.2%) 79(35.1%) 74(32.9%) 49(21.8%)
**Gender**	
Female Male	194(86.2%) 31(13.8%)
**BMI (mean ± SD**,** Kg/m**^**2**^**)**	
Normal (< 25 kg/m^2^) Overweight (25-<30 kg/m^2^) Obese (≥ 30 kg/m^2^)	29.1 ± 5.67 51(22.7%) 77(34.2%) 97(43.1%)
**Marital status**	
Married Other	176(78.2%) 49(21.8%)
**Education**	
Up to primary school Secondary school Diploma University and higher degree	55(24.4%) 95(42.2%) 41(18.2%) 34(15.1%)
**Employment**	
Employed Un-employed	37(16.4%) 188(83.6%)
**Family income (JD)**	
Less than 500 500 and above	121(53.8%) 104(46.2%)
**Living condition**	
With family Alone or others	210(93.3%) 15(6.7%)
**Smoking**	
Non or ex-smoker Current smoker	191 (84.9%) 34 (15.1%)
**Insurance status**	
Insured Non-insured	206 (91.6%) 19 (8.4%)
**How long do you have RA**	
Less than 5 years 5–10 years More than 10 years	103(45.8%) 53(23.6%) 69(30.7%)
**Number of chronic diseases**	
RA alone RA + one chronic disease RA + two chronic diseases RA + three or more chronic diseases	84(37.3%) 54(24%) 36(16%) 51(22.7%)
**History of DM**	
Yes No	54(24%) 171(76%)
**History of hypertension**	
Yes No	87(38.7%) 138(61.3%)
**History of other CVDs**	
Yes No	22(9.8%) 203(90.2%)
**History of thyroid disease**	
Yes No	28(12.4%) 197(87.6%)
**History of lung disease**	
Yes No	19(8.4%) 206(91.6%)
**History of dyslipidemia**	
Yes No	25(11.1%) 200(88.9%)
**History of osteoporosis**	
Yes No	56(24.9%) 169(75.1%)
**History of gout**	
Yes No	11(4.9%) 214(93.8%)
**History of osteoarthritis**	
Yes No	13(5.8%) 212(94.2%)

*Data are presented as frequency (%), mean ± standard deviation as appropriate. BMI: Body Mass Index, JD: Jordanian Dinar, DM: Diabetes Mellitus, CVDs: Cardiovascular Diseases, RA: rheumatoid arthritis

### RA-related medications used by the study sample

In this study, the most prescribed medication for RA participants was conventional synthetic disease-modifying antirheumatic drug (csDMARD) monotherapy, which included the single use of such csDMARD as methotrexate (MTX), leflunomide, hydroxychloroquine, or sulfasalazine. Based on Table 4, approximately 53% (*n* = 141) of the participants received csDMARD monotherapy. Also, csDMARD-bDMARD therapy, and double-csDMARD were only used by 19.1% (*n* = 43), and 11.6% (*n* = 26) of the study participants, respectively. In contrast, the use of biological monotherapy and triple-csDMARD therapy was less common, representing 4% (*n* = 4) and 0.9% (*n* = 2) of the study participants, respectively. Additionally, MTX was the most frequently prescribed csDMARD, representing 84.9% (*n* = 191) of the total study participants. Among the study participants, hydroxychloroquine was prescribed for 13.8% (*n* = 31), whereas 12.9% (*n* = 29) of them received sulfasalazine. Only 0.9% (*n* = 2) of the RA participants received leflunomide. The biological agents were prescribed for 22.66% (*n* = 51) of the study participants. According to our results, adalimumab was the most commonly prescribed TNF alpha inhibitor among 33.3% (*n* = 17 of the RA participants, followed by etanercept and golimumab, representing 27.45% (*n* = 14) and 15.68% (*n* = 8, respectively. However, 3.9% (*n* = 2 and 1.96% (of the study participants were on rituximab and tocilizumab, which are classified as non-TNF alpha therapies. Also, 9.8%(*n* = 5) were on tofacitinib, which is among the JAK inhibitors. Adjunctive therapy was used in the study. Approximately one-third (31.6%, *n* = 71) of the study participants received corticosteroids as adjuncts. Among the corticosteroids, prednisolone, with a dose of 5 mg per day, was the most frequently received. NSAIDs were used by 12.9% (*n* = 29), while 25.3% (*n* = 57) of the participants were on both corticosteroids and NSAIDs at the same time.

**Table 4:** Medication used in study population

**RA medications** Methotrexate Sulfasalazine Hydroxychloroquine Leflunomide TNF alpha Non TNF alpha JAK inhibitors	**Frequency (%)** 191(84.9%0 29(12.9%) 31(13.8%) 2(0.9%) 45(20%) 1(0.4%) 5(2.2%)
**Biological agents** Adalimumab Etanercept Golimumab Infliximab Rituximab Tocilizumab Tofacitinib	51 (22.66%) 17(33.3%) 14(27.45%) 8(15.68%) 4(7.8%) 2(3.9%) 1(1.96%) 5(9.8%)
**Drug categories used in patients**	
CsDMARD monotherapy Double-csDMARD therapy Triple-csDMARD therapy CsDMARD and bDMARD Bdmard	141(52.7%) 26(11.6%) 2(0.9%) 43(19.1%) 9(4%)
**Type of bridging therapy**	
Corticosteroids NSAIDs Both corticosteroids and NSAIDs	71(31.6%) 29(12.9%) 57(25.3%)
**Concurrent medications**	
Statins Gastroprotective agents CCBs Antidiabetics ACE inhibitors ARBs Beta blockers Diuretics Thyroid hormone Antibiotics Vitamins	35(15.6%) 109(48.4%) 22(9.8%) 37(16.4%) 17(7.6%) 33(14.7%) 36(16%) 16(7.1%) 19(8.4%) 6(2.7%) 203(90.2%)
**Number of medications**	
<5 ≥5	184(81.8%) 41(18.2%)

* Data are presented as frequency (%). RA: rheumatoid arthritis; TNF: tumor necrosis factor; Non-TNF: non tumor necrosis factor; JAK: Janus kinase inhibitors; bDMARD: biological disease-modifying antirheumatic drug; csDMARD: conventional synthetic DMARD; NSAID: nonsteroidal anti-inflammatory drug; CCBs: calcium channel blockers and diuretics; ARBs: angiotensin-receptor blockers, ACEs: angiotensin-converting enzyme

Multiple classes of supplements and medications were reported to be used by the study participants, including the following vitamins: folic acid, vitamin D3, and calcium carbonates. These drugs had been prescribed and implicated in 90.2% (*n* = 203) of the RA participants. Also, gastroprotective agents, including H2-receptor antagonists and proton-pump inhibitors, were prescribed to about half (48.4%, *n* = 109) of the RA participants. For cardiovascular diseases, most of the study participants (16%, *n* = 36) were on beta-blockers, followed by angiotensin-receptor blockers 14.7% (*n* = 33), angiotensin-converting enzyme inhibitors 7.6% (*n* = 17), calcium channel blockers 9.8% (*n* = 22), and diuretics 7.1% (*n* = 16). Antidiabetics were used in 16.4% (*n* = 37) of the study participants, and metformin was the most frequently used either as monotherapy or combination therapy. About 15.6% (*n* = 35) of the patients were on statins, 8.4% (*n* = 19) were on thyroid hormone, and only 2.7% (*n* = 6) of the study participants received antibiotics.

### Knowledge of rheumatoid arthritis [28]

The assessment of RA participants’ knowledge levels is shown in Table 5, along with the percentage of correct answers for each item. More than 50% of the study participants correctly answered 10 questions about their knowledge of RA, while more than 50% of them incorrectly answered 3 questions out of 13. RA participants had the highest correct response rate 97.8% (*n* = 220) for the question “Which of the following is a symptom of rheumatoid arthritis?” while the question “In your opinion, can rheumatoid arthritis spread from person to person?” was the second highest correctly answered at 88% (*n* = 198). In contrast, only 27.6% (*n* = 62) and 33.8% (*n* = 76) of the patients correctly answered, “Do you know what rheumatoid arthritis is?” and “Is physical therapy helpful in this disease?” respectively.

**Table 5:** Assessment of Disease Knowledge using RAKAS Scale

Item	The correct answer	Frequency (%) of correct answer
1.Do you know what rheumatoid arthritis is? -Yes, completely aware. -Yes, to some extent. -No.	Yes, completely aware Yes, to some extent	62 (27.6) 81 (37)
2.Which of the following is a symptom of rheumatoid arthritis? -Low blood sugar. -Joint pain. -High blood pressure. -Feeling sleepy.	Joint pain	220(97.8)
3.Which of the following is a risk factor of rheumatoid arthritis? -High blood pressure. -High blood sugar. -Presence of diabetes in parents. -Presence of rheumatoid arthritis in parents.	Presence of rheumatoid arthritis in parents	97(43.1)
4.In your opinion, dose rheumatoid arthritis only affects bones/joints? -Yes. -No. -I don’t know.	Yes	80(35.6)
5.In your opinion, can rheumatoid arthritis result in physical/work related disability? -Yes. -No. -I don’t know.	Yes	181 (80.4)
6.In your opinion, can rheumatoid arthritis result in deformity of bones /joints in the body? -Yes. -No. -I don’t know.	Yes	191(84.9)
7.In your opinion, can rheumatoid arthritis spread from person to person? -Yes. -No. -I don’t know.	No	198(88)
8.In your opinion, can rheumatoid arthritis be genetically inherited from parents? -Yes. -No. -I don’t know.	Yes	113(50.2%)
9.In term of gender, who is more prone to suffer from rheumatoid arthritis? -Male. -Female. -I don’t know.	Female	146(64.9)
10.which of the following lab test commonly used to evaluate RA? -ESR (Erythrocyte sedimentation rate). -Random blood sugar. -Blood pressure -Serum cholesterol	ESR	124(55.1)
11.In your opinion, is rheumatoid arthritis completely curable? -Yes. -No. -I don’t know.	No	146(64.9)
12.In your opinion, does it require lifelong treatment? -Yes. -No. -I don’t know.	Yes	164(72.9)
13.Is physical therapy helpful in this disease? -Yes. -No. -I don’t know.	Yes	76(33.8)
**Score of RAKAS** **Poor knowledge** (less than 30% correct answers = 4 points or less) **Low knowledge** (more than 30% or equal 50%correct answer = 5–7 points) **Adequate knowledge** (more than 50% or equal 75% correct answers = 8–10 points) **Excellent knowledge** (more than 75%correct answer = above 11points)		21(9.3) 47(20.9) 96(42.7) 61(27.1)

* Data are presented as frequency (%). * ESR: Erythrocyte sedimentation rate; RAKAS: rheumatoid arthritis knowledge assessment scale

According to RAKAS classification criteria, about a third of participants (27.1%, *n* = 61) had excellent knowledge about RA disease, with a score of 11 points or above out of 14, while 42.7% (*n* = 96) had adequate knowledge with a score of 8–10 points. However, only 30.2%(*n* = 68) of the RA participants had low to poor knowledge with a score of 7 points or less. Among the RA participants, knowledge was excellent (more than 75%) for four items [2, 5–7], adequate (more than 50–75%) for four items [8–11], low (more than 30–50%) for four items [1, 3, 4, 13], poor (less than 30%) for one item [1].

Based on Table 6, the results of the correlation analysis showed that the knowledge of disease positively correlated with the following parameters: income level, educational level, and duration of disease (*p* < 0.05). However, the knowledge of disease was negatively correlated with age (*p* < 0.05). Based on Table 6, gender was not correlated with the knowledge of disease (*p* < 0.05).

**Table 6:** Bivariate correlation of the factors associated with RAKAS score

Variable	Correlation coefficient	*P* value*
**Age**	-0.168	0.012
**Gender**	0.107	0.108
**Income level**	0.184	0.006
**Educational level**	0.402	0.000
**Duration of disease**	0.329	0.000

*Bivariate correlation. P-values < 0.05 were considered statistically significant, RAKAS: rheumatoid arthritis knowledge scale

Based on Table 7, the results of quantile regression showed that age was negatively associated with knowledge of RA disease. Regarding educational level, the up to primary school group had a significantly lower knowledge score than the secondary school or above group (B= -1.960% CI: -3.035 - -0.885, *p* value <0.05). Patients with a duration of RA less than 5 years had a significantly lower knowledge score than other groups (B= -2, 12% CI: -3.267 - -0.973, *p* value <0.05). According to the findings, however, RA knowledge was not significantly associated with gender or family income.

**Table 7:** Quantile regression results of the factors associated with RAKAS scale

Clinical variable	Quantile regression (B)	*P*-value*	95% confidence interval (CI)	
			Lower	Upper
**Age**	-0.08	0.000	-0.124	-0.036
**Gender**				
Male Female	-0.88 Reference	0.224	-2.301	0.541
**Family income (JD)**				
Less than 500 500 and above	-0.760 Reference	0.139	-1.77	0.250
**Educational level**				
-Up to primary school -Secondary school or higher	-1.960 Reference	0.000	-3.035	-0.885
**Duration of RA**				
Less than 5 years 5–10 years More than10 years	-2.12 -0.560 Reference	0.000 0.410	-3.267 -1.896	-0.973 0.776

*Quantile regression. P-values < 0.05 were considered statistically significant, RAKAS: rheumatoid arthritis knowledge scale; RA: rheumatoid arthritis; JD: Jordanian Dinar

### Quality of life (QoL)

The Kruskal-Wallis test was performed to assess the relationship between QoL and other variables due to a lack of normality assumption (see Table 1). After using Bonferroni correction, which adjusted the significance level across all comparisons to reduce the chances of Type I error, participants with a university degree (i.e., bachelor’s or higher) showed a significantly higher QoL than those who obtained a primary education degree (*p* < 0.01). Further, participants with remission/mild and moderate disease activity assessed by DAS28 reported a significantly higher QoL than those with severe disease activity (*p* < 0.001, *p* < 0.001, respectively). Furthermore, compared to participants with lower incomes, those with higher incomes had a higher level of QoL (*p* < 0.003).

Regarding the QoL assessment, the item with the highest response rate of “no problems” was “self-care” (63.1%). Conversely, the item with the lowest response rate for “no problems” was “pain/discomfort” (23.2%). The mean utility (± SD) value was 0.44(± 0.42). Based on Table 2, the linear regression model included the potentially predictable variables associated with the increased utility, the dependent variable. The regression model showed that the income variable, disease duration, and DAS score were significantly associated with the utility. Higher income levels were associated with an increase in utility, while lower DAS scores or lower duration of RA significantly predicted the increased utility among participants.

## Discussion

### Demographics

The current study revealed a significant predominance of female participants (86.2%) in the study sample. Similarly, a study by Cawley et al. (2023) showed a similar gender distribution among RA patients, with approximately 80% being female [29], which is consistent with the higher prevalence of RA in women, which has been widely described in the literature. For instance, Smolen et al. (2020) and Dougados, Kissel, et al. (2014) found that women are two to three times more likely to have RA than men [4, 30], which could be attributed to genetic, hormonal changes, and environmental factors [31]. This study highlights the impact of gender-specific management strategies in clinical practice. Consistent with prior research, this study found that women may experience more severe symptoms than men, demanding more intensive treatment approaches [32, 33]. Understanding these variations is critical for tailoring gender-specific interventions and improving patient outcomes.

The mean age of the study participants reflected the typical onset age for RA, which usually ranges between 40 and 60 years, which is consistent with the epidemiological data reported by Nygaard and Firestein (2020) and Smolen et al. (2020) [31, 34]. Age-related factors have significant implications for managing RA. Older patients are more likely to develop comorbidities such as cardiovascular disease, osteoporosis, and diabetes mellitus, which may influence their RA medication selection and susceptibility to adverse drug reactions [35]. The findings of Conway et al. (2014) stressed the need for age-specific comprehensive management plans [36].

### BMI and health status

The study’s findings showed that 22.7% of participants had a normal BMI, 34.2% were classified as overweight (BMI 25–29.9 kg/m²), and 43.1% were categorized as obese (BMI ≥ 30 kg/m²). Obesity and overweight may worsen RA symptoms and outcomes due to increased inflammation caused by pro-inflammatory cytokines secreted by adipose tissue (e.g., IL-6 and TNF-α) [37]. Furthermore, obesity is associated with several comorbidities, including diabetes, cardiovascular disease, and osteoarthritis, as well as a higher risk of disability and reduced physical function, all of which may contribute to poor RA treatment outcomes [38, 39].

### Marital status and socioeconomic factors

In this study, married individuals with RA reported better physical functioning and less disability compared to unmarried individuals, demonstrating how perceptions of disease activity and its consequences are positively influenced by marital status. Similar findings were also reported by Ulus et al. (2020) [40]. Further, social support can have a significant impact on health outcomes in chronic diseases, such as enhancing psychological well-being, providing emotional support, and encouraging better adherence to treatment regimens [41].

The distribution of the educational levels of the participants in this study highlights the diverse educational backgrounds of the RA population. Education is a significant determinant of health literacy and improves patients’ perceptions and management of their disease [42]. Enhanced health outcomes associated with higher levels of education could be attributed to individuals’ knowledge of the disease, their adherence to treatment plans, and their engagement in self-care practices [43]. A study by Taibanguay et al. (2019) indicated that RA patients with higher educational levels reported better health outcomes and increased adherence to treatment protocols, which supports the findings of this study [44]. Similarly, a study by Knudsen et al. (2024) revealed that tailored educational interventions significantly improved self-management and treatment adherence in RA patients [45]. Additionally, increased understanding can result in better employment prospects and better access to healthcare, which ultimately contribute to a higher quality of life [46].

The study found a high unemployment rate of 83.6%, with more than half of working individuals earning less than JD500 ($700) per month (53.8%), highlighting the economic burden of RA on patients. Unemployment and low-income levels are significant barriers to receiving medical care, medications, and support services [47]. Similarly, a study reported that unemployment rates among RA patients were significantly higher than the general population, which represents a major barrier to receiving the most effective care for the disease [48].

### Comorbid conditions

This study reported a high prevalence of chronic diseases among RA patients, including hypertension (38.7%) and diabetes (24%), which could be explained by the systemic inflammatory nature of RA [49]. According to previous research, the prevalence of diabetes and hypertension in RA patients was approximately 20% and 32%, respectively [50, 51]. Furthermore, a study reported hypertension rates of 49% and diabetes rates of 15% among RA patients, highlighting the prevalence of RA-related comorbidity in different countries and healthcare systems [52]. These findings emphasize the pressing need to recognize and alter cardiovascular risk factors early in RA disease to support lifestyle modifications and reduce the complexity of subsequent treatment regimens [13, 30].

### Knowledge assessment

While nearly two-thirds of the participants indicated adequate to excellent knowledge, a significant lack of awareness of the risk factors, disease characteristics, the impact of genetics, the required lab tests, and physical treatment in RA was uncovered. While the vast majority correctly identified the primary symptom of RA and that it is not transmissible from person to person, only 33.8% correctly identified physical therapy as a component of the treatment strategy. This gap emphasizes the need for health awareness programs to empower RA patients with health literacy and encourage the adoption of a healthy lifestyle. Similar findings were reported by Ndosi et al. (2016) and Joplin et al. (2015), indicating that RA patients struggled with inadequate literacy, understanding therapeutic strategies, and the long-term complications [53, 54].

### Factors affecting knowledge

The findings revealed that participants’ knowledge of RA was significantly associated with age, education level, RA duration, and income. Participants with secondary education or higher had significantly higher knowledge scores than those with only primary school (B= -2, 95% CI: -3.346 -0.654, *p* < 0.05). Patients with RA for over 10 years exhibited greater knowledge scores compared to those with shorter disease durations (B=-2.12, 95% CI: -3.267 -0.973, *p* < 0.05). Consistently, Verstappen (2015) found that patients with longer disease duration and higher education levels had a better understanding of their condition [55]. However, previous studies showed no correlation between disease knowledge and either disease duration or education level [56, 57], which could be attributed to the use of different measuring tools. In consistent with Knitza et al. (2020), who reported that younger patients had greater access to digital health resources, improving their disease knowledge [58]. This study confirmed that younger patients were more informed about their RA, possibly due to better access to information and higher health literacy rates. While other studies indicated no association between age and disease knowledge [57]. Gender differences were not statistically significant, as previously shown [59]. Although other studies, such as those by Townsend et al. (2014), Sokka et al. (2009), and Intriago et al. (2019) have noted that women often seek more information about their health conditions than men, potentially due to their higher involvement in healthcare decisions [60–62]. Income was also a crucial factor, as higher income levels were associated with better RA knowledge, possibly due to better access to healthcare resources and educational materials, as observed in studies by Quinlan et al. (2013) and Izadi et al. (2021) [63, 64]. The following study failed to find a correlation between income level and disease knowledge [57].

**Fig. 1:**
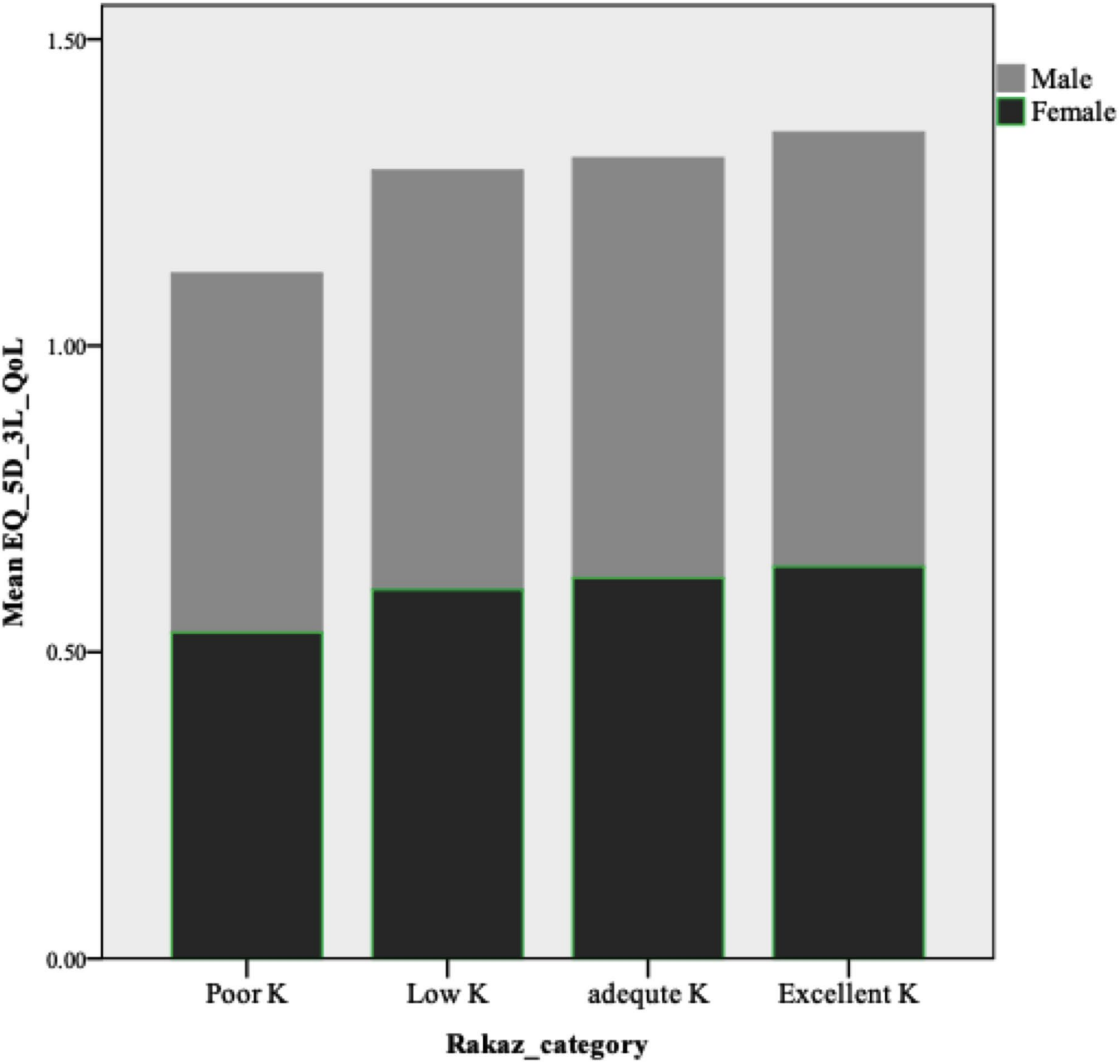
Although the relationship between more knowledge and better. quality of life (QoL) was not statistically significant; males had a slightly higher QoL than females

Based on the univariate and multivariable analysis, it has been revealed that the level of RA knowledge among participants has no significant relationship with the QoL based on the utility values (see Fig. 1). The empirical findings in this study confirmed a true relationship after including all the covariates in the regression models to check for confounders or interactions with the knowledge and to exclude any conditional independence relationship with a third variable. This might be attributed to the progressive nature and fluctuation in the disease activity. Further research is needed to assess the level of health literacy and self-awareness of the disease and its impact on lifestyle changes. Another explanation is that the RAKAS knowledge scale is not focused on patients’ disease management behavior, which may affect patients’ self-management in RA. Further, knowledge of comorbidities associated with RA [23, 30] may operate as a mediating factor between RA knowledge and QoL. The relationship between RA knowledge and QoL should be further investigated. On the other hand, several studies [65–67] found a significant correlation between knowledge and QoL among patients with chronic diseases, suggesting the role of knowledge in increasing the perceived risk of the disease and self-care strategies that help patients control their symptoms.

### Quality of life

The EQ-5D-3 L was used metric to obtain utility, which was its mean score of 0.62(± 0.22), potentially reflecting the disease activity, co-morbidities, and other contributing variables. The findings showed that participants with a bachelor’s degree or higher had significantly better utility than those with primary education (*P* = 0.007). Similarly, Gamal, Eleishi [68] and Jiang, Sandberg [69] found a strong relationship between higher education levels and increased health literacy, which strengthened patients’ ability to comprehend their conditions, follow treatment plans, and make well-informed choices for the betterment of health. Similarly, a study by López-Castillo, Calderón-Rojas [70] reported that RA patients with higher education could access and utilize healthcare resources more effectively. Moreover, Ndosi, Johnson [71] emphasized the role of education in enhancing self-efficacy, improving adherence to lifestyle modifications, and therefore enhancing QoL. In a similar vein, Kvien [72] suggested that educated patients are well-equipped to manage the long-term consequences of RA. Furthermore, a study by Putrik, Ramiro [73] showed that patients with higher education were more likely to improve their physical function and reduce pain levels through following health behaviors. However, another study observed a negative association between a high education level and quality of life [74].

Additionally, the Mann-Whitney U test and regression analysis showed a significant positive association between income and QoL in RA patients. This finding is consistent with the existing literature on the socioeconomic determinants of health, the key drivers of accessing medical services, medication affordability, and maintaining a healthier lifestyle. For instance, Baldassari, Cleveland [75] found that RA patients with higher incomes had better access to specialist care and disease-modifying antirheumatic drugs (DMARDs) with fewer delays in receiving treatment, leading to improved clinical outcomes and utility. Similarly, Wolfe, Häuser [76] demonstrated barriers to care, limited access to medications, and healthcare services among low-income groups. Studies by Abu Hamdeh, Al-Jabi [77] and Izadi, Li [78] showed that higher-income patients reported greater satisfaction with their care. While limited income may restrict access to healthcare and accelerate disabling disease progression [79]. Initiatives aimed at improving health literacy and self-care [80] should be encouraged to increase awareness about diet and exercise, which further enhances utility.

Additionally, the study findings indicated that income, disease activity, and disease duration were significant predictors of utility in RA patients. These results are consistent with the findings of Lapčević, Vuković [81], who reported that employment provides economic and social resources that, in turn, enhance physical and mental wellness. In a similar vein, Wan, He [82] concluded that financial stress and limited access to healthcare services were associated with unemployment. A study by Kwon, Rhee [83] found an association between unemployment and higher levels of anxiety and depression. Besides, its impact on social isolation and psychological well-being as reported by Holland and Collins [84]. Lastly, Verstappen [55] pointed out that better physical functioning and health-promoting behaviors among employed participants contributed to improved QoL.

Additionally, the study shows a strong negative relationship between DAS28 and QoL in RA patients, with higher DAS scores (indicating more severe disease activity) strongly linked to worse QoL (B = -0.133, *P* = 0.000). One-way ANOVA and post-hoc results showed that patients in remission or with mild to moderate disease activity had significantly better QoL than those with severe RA (*P* = 0.001). This aligns with evidence that remission or low disease activity improves physical function, reduces pain, and enhances mental health. At the same time, severe RA is linked to increased disability, chronic pain, and impaired mobility, all of which negatively impact daily functioning and QoL [85, 86]. Studies such as those by Matcham, Norton [87] confirm that patients with higher DAS scores experience more significant disease burden, including joint damage, fatigue, and mental health issues, all of which contribute to lower QoL.

Additionally, van Onna and Boonen [88] and Nurmohamed, Heslinga [49] have demonstrated that severe RA is associated with higher comorbidities, such as cardiovascular disease, further deteriorating QoL. Another study by Gettings [89] highlighted that sustained remission or low disease activity leads to significant improvements in both physical and psychological well-being, reinforcing the importance of achieving and maintaining low DAS scores in RA management. Lastly, Withers, Gonzalez [90] emphasized the critical role of early intervention and aggressive treatment in reducing DAS scores, which directly correlates with better long-term QoL outcomes for RA patients. These findings underscore that controlling disease activity is essential for improving QoL in RA patients, as higher DAS scores are closely tied to worse health outcomes.

Moreover, the findings of this study indicate that gender was not statistically significant with QoL. Gender was statistically rolled out as being mediating or moderating variable in predicting the QoL, which excluded the possibility of unequal distribution of contributing factors. In line with earlier research [91–93], subgroup analysis revealed that males reported fewer disease activities following anti-TNF medication (*p* < 0.05), but there was no gender-based variation in how they responded to non-biological DMARDs.

According to Scalone et al.(2013) [93], the Ceiling effect of the proportion of respondents reporting full health in this study was 19.6%, which is lower than what was reported by previous studies using EQ-5D-3 L [94, 95], indicating its high sensitivity and ability to detect deference within RA population. Further research is crucial to understand the disparities in the absolute and relative ceiling effect as well as the ceiling effect reduction using both the 5 L version and the 3 L version of EQ-5D.

### Clinical implications

The clinical implications of this study underscore the critical need for improved RA patients’ knowledge. Enhancing patients’ knowledge through tailored educational interventions is paramount, as it directly impacts patients’ ability to manage their condition effectively. Comprehensive care approaches, which integrate the management of comorbidities and leverage multidisciplinary care teams, are essential to address the complex needs of RA patients. By focusing on these areas, healthcare providers can significantly improve patient outcomes and ensure a holistic approach to RA management that encompasses medication optimization, patient education, and comprehensive care coordination.

### Limitations and strengths

The limitations of this study include the inability to demonstrate causality between variables due to the cross-sectional design and limited generalizability due to the relatively small sample size. Furthermore, the convenience of the sample collection method may increase the possibility of selection bias (e.g., sampling bias, non-response bias). While the study sample reflected some socioeconomic variables (e.g., income, insurance), there was a risk of selection bias based on disease severity, resulting in underrepresentation of less severe patients in the current study. As a result, multiple regression analysis was applied to minimize the impacts of various variables that might contribute to bias and influence the outcome variables. This potential bias should be addressed in a larger study with a more evenly distributed disease state among participants. Finally, the lack of cross-cultural validity for the RAKAS scale and the generic use of QoL measurement could potentially influence the study conclusion. Further, the adopted EQ-5D-3 L in this study may less effectively capture the utility values compared to the EQ-5D-5 L. However, the study’s strengths lie in its comprehensive approach, encompassing a wide range of demographic and clinical factors, and its high response rate of 94.9%, which enhances the reliability of the results. Also, the study utilized a validated instrument for assessing RA knowledge and used robust statistical methods to identify key factors impacting RA knowledge.

## Conclusion

This study concluded that most of the RA participants had adequate to excellent knowledge levels about their RA disease. While the current literature emphasized the role of inflammation control to achieve remission, the findings highlighted the significance of enhancing patient knowledge regarding RA and quality of life. The knowledge assessment tool found that participants had a mixed understanding of RA, with significant gaps in more detailed areas of symptom identification, disease treatment, and self-care. Further studies in the MENA region should enhance the cross-cultural validity of the RAKAS scale. The findings showed that younger patients, those with longer disease duration, higher education levels, and higher income had a substantial impact on better knowledge of RA. The need for educational interventions and awareness campaigns should be targeted towards promoting self-management behaviors. The findings also showed that the disease activity, low income, and longer disease duration significantly reduce patients’ quality of life, which can eventually limit patients’ access to healthcare facilities, seek advanced therapies, and live in balance with other comorbidities. Healthcare practitioners and policymakers can effectively enhance RA patient outcomes by incorporating comprehensive educational programs and tackling socioeconomic constraints. This study lays the ground for future research to further assess the determinants of quality of life among patients with RA.

While the male-female ratio in this study may limit the generalizability of the current findings and weaken the true moderating effect of genders on outcome variables (e.g., DAS28, RAKAS), as the majority of participants at the recruitment site had higher disease activity, which was the primary reason for the clinic visit. A more gender-balanced sample could better predict treatment responses based on gender and reduce the chance of selection bias. The varying prevalence of comorbidities, treatment options (such as anti-TNF vs. cDMARDs), and risk factors for disability-adjusted life years (DALYs) may have an impact on patients’ reported outcome measures, which could explain this conclusion. However, compared to genetic and hormonal factors, the unequal distribution of the statistically controlled attributing factors between genders may have a smaller impact on disease activity [96].

## Data Availability

Data are available upon reasonable request.
